# Quality of sleep in individuals with systemic sclerosis and its correlation with functional disability and quality of life: a cross-sectional study

**DOI:** 10.1590/1806-9282.20231254

**Published:** 2024-05-03

**Authors:** Gabriela da Silva Santos, Marcella Ferreira Barros, Daniel Neri da Matta, Angélica da Silva Tenório, Rafaela Silva Guimarães Gonçalves, Angela Luzia Branco Pinto Duarte, Andréa Tavares Dantas

**Affiliations:** 1 Universidade Federal de Pernambuco, Department of Physiotherapy – Recife (PE), Brazil.; 2 Universidade Federal de Pernambuco, Department of Clinical Medicine – Recife (PE), Brazil.

**Keywords:** Scleroderma, systemic, Sleep quality, Daytime sleepiness, Quality of life

## Abstract

**OBJECTIVE:**

This study aimed to evaluate the quality of sleep in individuals with systemic sclerosis and its correlation with the quality of life and disability.

**METHODS:**

This is a cross-sectional study, carried out in a tertiary service of a university hospital. Inclusion criteria were diagnosis of systemic sclerosis according to the criteria of the American College of Rheumatology/European League Against Rheumatism 2013 or the preliminary criteria of the American College of Rheumatology 1980, age ≥ 18 years; regularly monitored at the outpatient clinic of rheumatology. Clinical and demographic data of the patients were obtained through a structured interview and evaluation of the medical records. Sleep quality was assessed using the Pittsburgh Sleep Quality Index questionnaire, daytime sleepiness using the Epworth Sleepiness Scale, quality of life using 12-item short-form health survey, and disability using the scleroderma health assessment questionnaire.

**RESULTS:**

A total of 50 patients with systemic sclerosis were included, with 92% female, mean age 48.9 years, mean disease duration 8.9 years, and 60% limited cutaneous form. Most systemic sclerosis patients (84%) have poor sleep quality and 20% have excessive daytime sleepiness. There was a significant negative correlation between Pittsburgh Sleep Quality Index and the physical and mental components of the 12-item short-form health survey (r=-0.42, p=0.003 and r=-0.43, p=0.002, respectively) and a positive correlation with the scleroderma health assessment questionnaire (r=0.52, p=<0.001).

**CONCLUSION:**

This study showed that poor sleep quality is a very common finding among systemic sclerosis patients, and it negatively affects both the quality of life and the degree of disability.

## KEYPOINTS

Sleep quality is an unmet need in patients with systemic sclerosis

Poor sleep quality is very common in patients with systemic sclerosis

Poor sleep quality correlated with worse quality of life and greater disability

## INTRODUCTION

Systemic sclerosis (SSc) is a rare autoimmune disease characterized by vascular involvement, autoimmunity, and progressive fibrosis of the skin and internal organs. Given its heterogeneous clinical manifestations and its chronic and progressive character, patients (SSc) have significant functional impairment and quality of life. While the traditional medical approach has generally focused on treating target organ manifestations such as in the skin, lungs, and heart, patients may perceive other manifestations as more important or debilitating. Factors such as fatigue, depression, dissatisfaction with their body image, joint problems, sexual dysfunction, sleep disturbances, and pruritus are some obstacles that these patients face to fully achieve well-being^1,2^. Thus, the purpose of treatment should be to maintain control of the disease and to ensure improvement in the quality of life through a comprehensive analysis of the patient and multidisciplinary support^3^.

Sleep quality is an important component of quality of life, but the impact of SSc on the sleep of affected individuals is still a poorly studied issue^4-6^. In a large Canadian cohort, difficulty sleeping was reported as one of the five highest-rated symptoms in terms of frequency and moderate-to-severe impact on daily activities^2^. A study with polysomnography showed changes such as reduced sleep efficiency and increased amount of periodic limb movements^7^, while another study showed a high frequency of obstructive sleep apnea in patients with SSc^8^. Possible causes of sleep disorders in SSc include fatigue, functional limitations, skin deformities, pain, restless legs syndrome, dyspnea, gastroesophageal reflux, and psychological disorders such as depression^6^. Therefore, the objective of this study was to evaluate the quality of sleep in individuals with SSc and its correlation with the quality of life and disability.

## METHODS

### Patients

This is a cross-sectional study which was approved by the local ethics committee. Data were collected from 50 patients with SSc treated at the Rheumatology Service of the Hospital das Clínicas of the Federal University of Pernambuco (HC-UFPE), who were selected according to outpatient care using the eligibility criteria.

Inclusion criteria were as follows: over 18 years of age; diagnosis of SSc according to the preliminary criteria of the American College of Rheumatology^9^ or the criteria of the American College of Rheumatology/European League Against Rheumatism (ACR/EULAR) 2013^10^; and regularly monitored at the outpatient clinic of rheumatology at HC-UFPE. Exclusion criterion was diagnosis of localized scleroderma or known diagnosis of mental retardation or dementia.

Demographic and clinical information were obtained from an interview of the patients by a trained evaluator, and then the questionnaires were applied. Complementary information regarding the diagnosis, complementary exams, and treatment employed was obtained from medical records.

The study was approved by the Committee on Ethics in Research of Human Beings UFPE (CAAE- 77235517.8.1001.5208) in accordance with the precepts of the Brazilian Health Council. Informed consent was obtained from all individual participants included in the study.

### Assessment instruments

Sleep quality was evaluated using the Pittsburgh Sleep Quality Index (PSQI) questionnaire, which was developed in 1989^11^ and assesses sleep quality in a standardized questionnaire, which can be easily answered and is validated for Brazilian Portuguese^12^. It consists of 19 questions answered by self-report and 5 answered by a roommate if they have one. The instrument assesses 7 sleep components: subjective sleep quality, sleep latency, sleep duration, habitual sleep efficiency, sleep changes, use of sleep medication, and daytime sleep dysfunction. The score ranges from 0 to 3 for each component, with a maximum score of 21 points. Scores ≥ 5 indicate poor sleep quality^13^. The Epworth Sleepiness Scale (ESS-BR) was used to assess daytime sleepiness. The questions are answered through self-report involving six daily situations and the chance for the patient to fall asleep when performing them. The score ranges from 0 to 18, and a score greater than 10 indicates excessive daytime sleepiness^13^.

Quality of life assessment was performed using the 12-Item Short-Form Health Survey (SF-12) instrument, a questionnaire consisting of 12 questions and has two domains: physical and mental^14^. The values for each domain are transformed into a scale ranging from 0 to 100, where 0 is equivalent to a worse quality of life and 100 to a better quality of life^15^. Functional disability was assessed using scleroderma health assessment questionnaire (SHAQ)^16^. It consists of 20 items spread over eight domains together with five additional domains that assess the dysfunctions caused by the symptoms of the disease. A visual analog scale is used to evaluate the additional domains. The values of each domain vary from 0 to 3, and the total score is obtained after adding all the values and dividing the total by 13^16^.

### Statistical analyses

The data collection took place from August 2019 to March 2020. The collected data were organized in the Excel XP 2016 Microsoft^®^ spreadsheets and analyzed using the GraphPad Prism 6.0 software program. Descriptive statistics were performed using mean and standard deviation for variables with normal distribution, a median and interquartile range for those with non-normal distribution, and frequency (percentage) for qualitative variables. Possible differences between means in the intergroup analysis were verified using the Student's t-test for independent samples when the sample presented normal distribution and Mann-Whitney test in cases of non-Gaussian distribution. Pearson's (samples with normal distribution) or Spearman's correlation test (samples with non-Gaussian distribution) was used to assess the relationship between two continuous variables. p<0.05 were considered significant. For correlation magnitude, it was considered that 0.1 is a small magnitude, 0.5 is a medium magnitude, and 0.8 is a large magnitude^17^.

## RESULTS

The demographic and clinical characteristics of the assessed patients are described in Table 1. About 42 patients (84%) had a PSQI score ≥ 5, indicating poor sleep quality, and 10 patients (20%) had excessive daytime sleepiness (ESS-BR>10).

**Table 1:** Demographic and clinical characteristics of patients with systemic sclerosis (n=50).

Sociodemographic and clinical characteristics		
Age (years) median (IQR)		52.0 (37.7–58.2)
Female n (%)		46 (92%)
Disease duration (years) median (IQR)		7.0 (4.0–13.0)
Clinical subset n (%)		
	Limited cutaneous	30 (60%)
	Diffuse cutaneous	18 (36%)
	Sine scleroderma	2 (4%)
	Overlap	6 (12%)
Clinical manifestations n (%)		
	Raynaud's phenomenon	49 (98%)
	Interstitial lung disease	34 (68%)
	Esophageal dysmotility	39 (78%)
	Digital ulcers	26 (52%)
	Myopathy	5 (10%)
	Pulmonary arterial hypertension	5 (10%)
	Arthritis	34 (68%)
	Telangiectasia	15 (30%)
	Calcinosis	5 (10%)
PSQI mean (±SD)		9.63 (±4.88)
ESS-BR mean (±SD)		6.62 (±5.23)
SHAQ mean (±SD)		1.42 (±0.73)
SF-12 PCS mean (±SD)		35.36 (±10.28)
SF-12 MCS mean (±SD)		42.60 (±12.38)

ESS-BR: Epworth Sleepiness Scale–Portuguese version; IQR: interquartile range; MCS: mental component summary; PCS: physical component summary; PSQI: Pittsburgh Sleep Quality Index; SD: standard deviation; SF-12: 12-item short-form health survey; SHAQ: scleroderma health assessment questionnaire.

Table 2 presents the correlations between sleep quality and the outcomes of disability and quality of life. It can be seen that there was a significant negative correlation between the components of the SF-12 and the PSQI and a significant positive correlation between SHAQ and PSQI. No associations were observed between sleep quality and demographic characteristics or clinical manifestations (Table 3).

**Table 2:** Correlation between sleep quality and age, disease duration, disability, and quality of life in patients with systemic sclerosis.

	Sleep quality (PSQI)		
	R	95%CI	p-value
Age	-0.09	-0.37 to 0.20	0.53
Disease duration	-0.03	-0.33 to 0.28	0.86
ESS-BR	-0.09	-0.37 to 0.20	0.55
SHAQ	0.52	0.26 to 0.70	<0.001
SF-12 PCS	-0.42	-0.63 to −0.14	0.003
SF-12 MCS	-0.43	-0.64 to −0.16	0.002

ESS-BR: Epworth Sleepiness Scale–Portuguese version; MCS: mental component summary; PCS: physical component summary; PSQI: Pittsburgh Sleep Quality Index; SF-12: 12-item short-form health survey; SHAQ: scleroderma health assessment questionnaire.

**Table 3:** Associations of sleep quality Pittsburgh Sleep Quality Index with the clinical characteristics in patients with systemic sclerosis.

Characteristic	Status	Mean PSQI (±SD)	p-value
Sex	Female	9.9 (±4.8)	0.18
	Male	6.5 (±4.8)	
Clinical subset	Limited	9.9 (±4.9)	0.69
	Diffuse	9.4 (±5.1)	
Interstitial lung disease	Yes	10.2 (±5.1)	0.24
	No	8.4 (±4.2)	
Esophageal dysmotility	Yes	10.0 (±5.0)	0.30
	No	8.2 (±4.3)	
Digital ulcers	Yes	10.5 (±4.9)	0.17
	No	8.6 (±4.7)	
Pulmonary arterial hypertension	Yes	11.0 (±6.6)	0.51
	No	9.5 (±4.7)	
Myopathy	Yes	8.4 (±3.9)	0.56
	No	9.8 (±5.0)	
Arthritis	Yes	10.1 (±4.9)	0.35
	No	8.7 (±4.9)	

## DISCUSSION

In the present study, we showed that most patients with SSc have poor sleep quality, which in turn was associated with the worse quality of life and greater disability. Although it is an important aspect related to the quality of life and a frequent complaint among patients, few previous studies have evaluated the sleep quality in individuals with SSc^1-9^. Sleep assessment in patients with autoimmune rheumatic diseases has been shown to be extremely important. In addition to the recognized impact on fatigue, mood, productivity, and quality of life, sleep disturbances have been associated with increased systemic inflammation and greater sensitivity to pain. Furthermore, recent studies have suggested an association between sleep disorders and an increased risk of chronic diseases, including autoimmune diseases^18,19^.

Considering a PSQI cutoff of 5, we observed that most patients with SSc (84%) had poor sleep quality. The mean PSQI score in our patients was high, similar to that described in a study with Italian^20^ and Brazilian patients^4^, and higher than that described in Turkish patients^6^. Although our study did not evaluate a control group, these previous studies had already shown that SSc patients had higher PSQI scores compared with healthy individuals and patients with rheumatoid arthritis^6,20^.

We found a significant correlation between disability and quality of life with sleep quality. These findings are in line with those described by other authors, who also found a correlation between sleep quality and disability and worse quality of life^4,6^. This finding is probably because quality sleep is an important component of quality of life, so poor sleep quality can affect this perception and other symptoms such as pain and fatigue that influence disability.

Although previous studies have shown an association between sleep disturbance and clinical manifestations of the disease, such as dysphagia, gastroesophageal reflux, pulmonary impairment, and pain^4,6^, we were unable to establish any association between these variables. In this study, the assessment of clinical manifestations was made according to the medical records, which did not necessarily reflect the activity of these manifestations. Therefore, it is possible that the lack of association between sleep quality and clinical manifestations of SSc is due to the absence of disease activity and good control of symptoms related to esophageal or pulmonary involvement. Furthermore, it is possible that sample size and demographic and clinical differences between patients in different studies may also be implicated.

Excessive daytime sleepiness can have an important impact on quality of life and functional impairment, in addition to being associated with the risk of morbidity and mortality related to cardiovascular, psychiatric, and neurodegenerative pathologies. Among the associated causes, sleep deprivation, obstructive sleep apnea, psychiatric or central nervous system disorders, and side effects of medications were common^21^. Taylor-Gjevre et al., evaluated a sample of patients with different rheumatic diseases and found excessive sleepiness in about 25.7% of patients and demonstrated a correlation with fatigue, quality of life, and disability^22^. In the present study, 20% of patients with SSc had excessive daytime sleepiness, but we did not observe an association with sleep quality or with the other clinical parameters evaluated.

This study has some limitations. The small sample size may have limited the detection of associations between sleep quality and clinical manifestations of the disease. Factors such as pain, anxiety, depression, and fatigue, in addition to more objective sleep assessments such as polysomnography, were not evaluated. Furthermore, the cross-sectional design of our study does not enable establishing causal relationships between the studied variables. Therefore, prospective studies with a larger number of patients are needed to better assess the factors that should be modified to improve sleep quality in patients with SSc.

In summary, this study highlights the high percentage of patients with compromised sleep quality and the high frequency of excessive daytime sleepiness in patients with SSc, reinforcing the importance of assessing sleep health in patients with SSc. Depending on the underlying cause, interventions such as guidance on sleep hygiene, weight loss, adjustment of medications in use, and control of predisposing factors, such as pain and gastroesophageal reflux, may be sufficient to achieve better sleep quality. In more specific situations, evaluation by a sleep specialist may be necessary^21^.

## CONCLUSION

This study showed that poor sleep quality is a very common finding among SSc patients. It also demonstrated that poor sleep quality negatively affects both the quality of life and the degree of disability of individuals with scleroderma. Therefore, we emphasize this as an important aspect to be evaluated and treated in patients with SSc.
